# Aortic Regurgitation in Bicuspid Aortic Valve: The Role of Multimodality Imaging

**DOI:** 10.3390/jcm13133924

**Published:** 2024-07-04

**Authors:** Lucia La Mura, Maria Lembo, Francesca Musella, Marianna D’Amato, Antonello D’Andrea, Raffaele Izzo, Giovanni Esposito

**Affiliations:** 1 Department of Advanced Biomedical Sciences, University Federico II of Naples, 80131 Naples, Italy; maria.lembo@unina.it (M.L.); rafizzo@unina.it (R.I.); espogiov@unina.it (G.E.); 2 Division of Cardiology, S. Maria delle Grazie Hospital, 80078 Pozzuoli, Italy; francmusella@gmail.com; 3 Division of Cardiology, Department of Medicine, Karolinska Institutet, 17177 Stockholm, Sweden; 4 Servicio de Cardiologìa, Hospital Central de La Defensa Gomez Ulla, 28028 Madrid, Spain; marianna.damato89@gmail.com; 5 Department of Cardiology, Umberto I Hospital, 84014 Nocera Inferiore, Italy; antonellodandrea@libero.it

**Keywords:** bicuspid aortic valve, chronic aortic regurgitation, multimodality imaging, echocardiography, computed tomography, magnetic resonance imaging

## Abstract

The evaluation of aortic regurgitation (AR) in bicuspid valve (BAV) is still a challenge because of the eccentricity of the jet, which may under/overestimate the regurgitation. The commonly used echocardiography parameters (such as vena contracta, pressure half-time, etc.) may not be useful in this kind of patient. A multimodality approach combining echocardiography, cardiac MRI, cardiac CT, and advanced technologies applied to non-invasive cardiac imaging (e.g., 4D flow and strain imaging) may be useful to better quantify regurgitation and to select patients suitable for valve replacement. This review provides an overview of the most recent insights about cardiovascular imaging tools and their utility in BAV evaluation, focusing on chronic regurgitation. We describe the role of multimodality imaging in both diagnosis and risk assessment of this disease, pointing out the advantages and disadvantages of the imaging techniques, aiming to provide a guide to clinicians and cardiovascular imaging specialists in choosing the best imaging tools to use.

## 1. Introduction

Bicuspid aortic valve (BAV) is the most common congenital heart disease, with a reported prevalence of 0.4–2.25% [1]. It is considered to be a valvulo-aortopathy characterized by significant heterogeneity of its valvular and aortic phenotypic expressions, associated disorders, complications, and prognosis. The most frequent complications of the BAV condition are aortic valvular dysfunction and ascending aorta (AAo) dilation. Early and accurate diagnosis of BAV using imaging techniques is essential to better manage follow-up and improve patient prognosis. Regarding valvular dysfunction, the evaluation of aortic regurgitation (AR) in BAV is still a challenge because of the eccentricity of the jet, which may under/overestimate the regurgitation. Transthoracic echocardiography (TTE) is used as first-line imaging, but the common echocardiography parameters (such as vena contracta, pressure half-time, etc.) are not often useful in this kind of patient. The use of a multimodality approach that combines echocardiography, cardiac MRI (CMR), cardiac CT, and advanced technologies (4D flow, strain imaging, etc.) may be useful to better quantify regurgitation and to select patients suitable for valve replacement.

### 1.1. Normal Aortic Valve Anatomy and Nomenclature

The aortic valve is one of the four cardiac valves. It is located in the aortic root, dividing the outflow tract of the left ventricle (LVOT) from the aorta. Its parts include the Valsalva sinuses, the fibrous interleaflet triangles, and the valve leaflets themselves. The three Valsalva sinuses are the areas between each leaflet and the inner surface of the aortic root wall, creating three bulges, each corresponding to a respective sinus [1]. The ridge at the top of the sinuses forms a distinct circular ring composed of thickened aortic wall tissue, known as the sinotubular junction, which marks the transition point from the aortic root to the AAo. The Valsalva sinuses are named according to the coronary artery whose opening is located within or near each sinus (right, left, and non-coronary). The three leaflets of the aortic valve are attached along the entire length of the root in a crescent shape. Due to this crescent-shaped attachment of the aortic valve leaflets, there are three triangular extensions of the left ventricular outflow tract that extend up to the sinotubular junction [2].

### 1.2. Classification and Phenotypes of BAV

**Figure 1:**
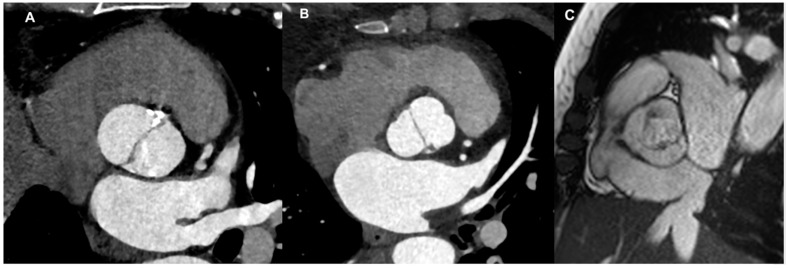
Different BAV types: (**A**) Sievers type 0 (valve with no raphe) by CT scan; (**B**) Sievers type 1 (valve with only one raphe) by CT scan; (**C**) partial-fusion BAV by CMR cine-sequence.

There are multiple nomenclatures and classifications for the BAV condition [3,4]. The most common used is Sievers classification system, in which three mayor types were identified: type 0 (no raphe in the valve) (Figure 1A), type 1 (only one raphe in the valve) (Figure 1B), and type 2 (two raphes in the valve). Based on this classification, the most prevalent type is type 1, representing approximately 90% of patients [5]. Depending on the position of the raphe in relation to the coronary sinuses, types 1 and 2 are categorized as left (L), right (R), and none (N) types. A new nomenclature/classification system was published, derived from an international consensus statement and representing the combined efforts of international BAV experts [4]. According to this further classification, three BAV types are identified—the fused BAV, the two-sinus BAV, and the partial-fusion BAV (Figure 1C)—each with different phenotypes [6]. The fused BAV is the most common, accounting for about 90–95% of cases [3]. It is characterized by two of the three cusps appearing fused or joined within three distinguishable aortic sinuses, resulting in two functional cusps (one fused or conjoined and the other non-fused) that typically vary in size and shape. Within the fused type, there are three specific BAV phenotypes: right–left cusp fusion, right–non (non-coronary) cusp fusion, and left–non (non-coronary) cusp fusion. The right–left cusp fusion phenotype is the most common, occurring in 70–80% of American, European, and Asian populations [3].

### 1.3. BAV Syndromes and Associated Cardiovascular Malformations

BAV is transmitted as an autosomal dominant trait with incomplete penetrance and variable expressivity due to a complex genetic structure involving many interacting genes [7]. It is usually an isolated defect, but 20% and possibly up to 50% of BAV patients have additional congenital cardiovascular malformations. Due to the shared embryologic origin of the aortic valve, LVOT, and proximal aorta, BAV often coexists with other left-sided congenital heart defects, such as coarctation (CoA), Shone complex, and hypoplastic left heart syndrome. Approximately 50 to 85% of patients with CoA have BAV [8]. Subjects with both CoA and BAV are more likely to develop valvular dysfunction and aortic aneurysm [9]. In addition, congenital coronary anomalies have been variably found in the BAV condition [10]. BAV is also associated with some genetic syndromes: Turner’s syndrome, characterized by complete or partial absence of one X chromosome, where BAV is likely the most common cardiovascular malformation (up to 30% of cases) [11]; Loeys–Dietz syndrome, caused by dominantly inherited mutations of TGF-β pathway genes (approximately 10% of cases) [12]; velocardiofacial syndrome (about 3%) [13]. It is always better to carry out a screening to exclude a BAV in the presence of physical alterations or aortic dilatation suggestive of the aforementioned syndromes.

### 1.4. Prognosis of BAV

BAV patients have high incidence of valvular and aortic complication occurrence, with a prevalence of AAo dilation ranging from 20% to 84%, depending on the study population [14].

The International Consensus recognizes three types of BAV-associated aortopathies—the ascending phenotype, root phenotype, and extended phenotype [4]. The root phenotype, characterized by aortic dilation mainly at the sinuses with a normal or less dilated ascending tract, may indicate more severe aortopathy, necessitating closer monitoring and earlier intervention. The more common ascending phenotype has been shown to be a more stable condition, typically exhibiting slower progression [15].

Lopez et al. observed that the mean annual growth of the aortic root is 0.15–0.23 mm/year, and a rapid aortic root dilation rate (>0.35 mm/year) is associated with hypertension, presence of raphe, AR, and male sex [16]. Anyway, aortic dissection represents the most feared complication, with a reported incidence of 3.1 cases per 10,000 patient-years (eight times the incidence in the general population), increasing to 0.5% in patients with aortic diameters > 45 mm [17]. Current guidelines recommend aortic surgery in BAV patients with an aortic diameter ≥ 55 mm and in patients with BAV, an aortic diameter ≥ 50 mm and concomitant risk factors, such as aortic coarctation, family history of dissection, arterial hypertension, or increase in aortic diameter 0.3 mm per year [18].

Regarding valve dysfunction, aortic valve replacement occurs in over 50% of BAV patients and it is recommended in symptomatic patients with severe AR and asymptomatic with LV dilation and systolic dysfunction. Young patients are more likely to develop AR, while elderly patients more likely aortic stenosis [18], with a prevalence of valve dysfunction between 47% and 64% and moderate-to-severe grade in less than 30% [14,19]. The most common causes of chronic insufficiency are annular dilatation and cusp prolapse or retraction, which may occur alone or in combination [20].

In addition, the anatomical anomaly predisposes the valve to an increased risk of endocarditis with an incidence of 9.9 per 10,000 patient-years [21].

Once a BAV has been diagnosed, it is important to educate the patient about the risks related to the progression of the disease and the importance of screening first-degree relatives, especially in the presence of AAo aneurysm or dissection familial history [22].

## 2. Assessment of Chronic AR in BAV: Usefulness and Limitations of Echocardiography

Echocardiography represents the primary diagnostic tool for the evaluation of both AR and stenosis, conditions potentially often present and concomitant in BAV patients, due to its high availability, low cost, and absence of radiation exposure. It allows for a multiparametric quantification of AR, with quantitative and semi-quantitative tools. The main limitation is given by the acoustic window, the extreme eccentricity of the jet and the irregular shape of the regurgitant orifice.

### 2.1. Qualitative and Quantitative Assessment

**Figure 2:**
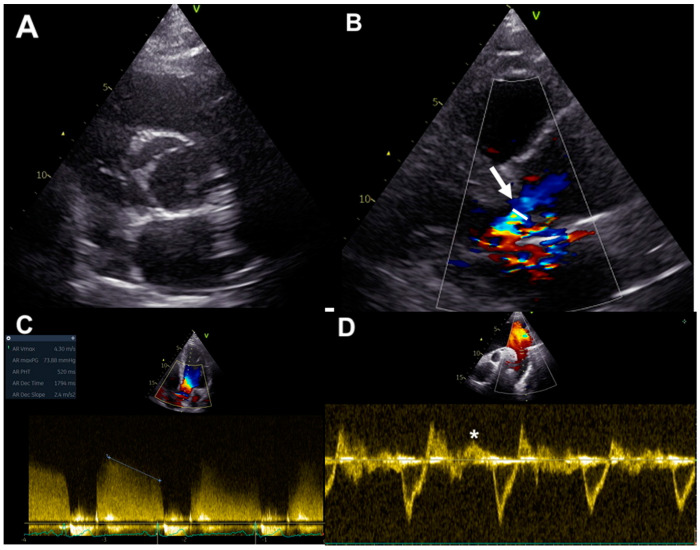
BAV assessment by echocardiography: (**A**) valve anatomy in parasternal short axis; (**B**) vena contracta width in parasternal long-axis (arrow); (**C**) pressure half-time measurement in continuous-wave Doppler; (**D**) assessment of the diastolic flow reversal velocity in the descending aorta by pulsed-wave Doppler (*).

Ultrasound imaging permits the evaluation of cusps morphology, commissure variations, the presence and location of a raphe and root structure, providing information about aortic valve anatomy, and the mechanisms underlying valve regurgitation [23] (Figure 2A). Assessment of AR severity in BAV patients relies on color, continuous- and pulsed-wave Doppler measurements of the aortic regurgitant jet, together with the evaluation of left ventricular (LV) volumes and function, as for tricuspid valve patients [24]. Considering valve anatomy and the often eccentric and irregular shape of BAV jet regurgitations, some methodologies for the evaluation of AR severity grading are more advisable in BAV patients. Color flow Doppler imaging allows for visual estimation of the aortic regurgitant jet and gives information about jet eccentricity, a condition often present in BAV. The diameter and the cross-sectional jet area at its origin are semi-quantitative color Doppler indexes of AR severity. Since BAV patients often have an irregular shape of the regurgitant orifice, the ratio between the regurgitant jet width and LVOT diameter should not be used in this setting [23]. On the other hand, vena contracta width, representing the smallest flow diameter at the level of the aortic valve in LVOT, just below the flow convergence site, can be employed for the estimation of AR in BAV, being applicable even in eccentric regurgitation jets; AR is mild up to the limit of 3 mm and severe beyond the limit of 6 mm [25] (Figure 2B). Three-dimensional echocardiography was demonstrated to be a useful tool for the visualization of the actual shape of the regurgitant aortic orifice, and for the evaluation of the area of vena contracta, which correlates well with the effective regurgitant orifice area (EROA) [26]. Nonetheless, it has to be considered that the vena contracta assessment has the limitation to not be applicable in the presence of multiple regurgitation jets [24].

Furthermore, the proximal isovelocity surface area method can be used in BAV patients for the assessment of AR severity, allowing for estimation of EROA and regurgitant volume (RV). Using this methodology, AR severity grading is classified on the basis of EROA and/or RV, with severe AR defined for an EROA ≥ 30 mm^2^ or an RV ≥ 60 mL. However, this technique is limited by low feasibility in a big proportion of patients because of difficulty in detection of the flow convergence zone and possible interposition of valve tissue [24].

Continuous-wave Doppler-derived pressure half-time of regurgitant aortic jets could be used in BAV patients, but this technique requires an adequate Doppler angle for minimizing errors related to non-correct ultrasound beam alignment and this could be often difficult or not feasible, in particular in eccentric regurgitation jets. A pressure half-time less than 200 ms is associated with severe AR [24] (Figure 2C).

In addition, the assessment of the diastolic flow reversal in the descending aorta by pulsed-wave Doppler represents a strong parameter for evaluating AR severity: severe regurgitation is defined for holodiastolic flow reversal at velocities more than 20 cm/s [24] (Figure 2D).

### 2.2. LV and Aortic Size Evaluation

Chronic AR in BAV patients could impact on LV morphology and function, determining eccentric hypertrophy, dilation, and systolic dysfunction over time. The evaluation of LV size and function by echocardiography is also used to guide the timing of intervention and to address patients to surgery [27]. Furthermore, ultrasounds are also useful for investigating the feasibility of valve-sparing aortic surgery or valve repair. Current guidelines recommend surgery in symptomatic patients with severe AR and low operative risk, regardless of the left ventricular ejection fraction (LVEF) value. Surgery is also recommended in asymptomatic patients with an LV end-systolic diameter > 50 mm (or >25 mm/m^2^ BSA) or resting LVEF ≤ 50% [18]. Therefore, transthoracic echocardiography allows for the first diagnostic approach for the evaluation of BAV aortopathy, providing information about the aortic diameters at different levels; however, this methodology has the limitation of being unable to evaluate the entire ascending and descending aorta.

### 2.3. Strain Imaging

Speckle-tracking echocardiography (STE) has emerged in recent years as a reliable and accurate measure of LV systolic function [28] being rapid, angle-independent, and calculable offline [29]. It can also be used to assess the elastic properties of arterial walls [30] by calculating the circumferential and longitudinal deformation they undergo during the cardiac cycle.

Patients with BAV and impaired LV global longitudinal strain (GLS), as a subclinical myocardial damage parameter, may have more advanced stages of valvular disease and may develop symptoms or early LV systolic dysfunction, and therefore be referred for surgery sooner [28].

Aortic longitudinal strain was suggested to be a good parameter to evaluate aortic distensibility. Analyzing aortic GLS values throughout the increment in AR severity, it decreases from mild to moderate AR while it increases exponentially in severe AR; moreover, the measurement is not reliable for very high aortic diameters [31].

Nowadays, only few data are available on the usefulness of these new parameters and further studies are needed.

## 3. Assessment of Chronic AR in BAV: Usefulness and Limitations of CMR

In recent years, the use of CMR in the evaluation of valve regurgitation has become more widespread, despite the lesser availability of instrumentation and the limitation of the high acquisition time. The calculation of the regurgitant volume (RV) represents the most reliable and reproducible quantitative method even in the case of eccentric jets; however, it is affected by the orientation of the acquisition plane and a low temporal resolution. CMR is also the gold standard for the evaluation of ventricular volumes and function as well as allowing for tissue characterization through LGE and T mapping sequences.

### 3.1. Quantitative Assessment

A major advantage of CMR is the ability to assess and quantify AR in an easy and reproducible manner. AR may appear as one or more flow jets, typically holodiastolic, originating from the aortic valve and directed to the LVOT, easily identified in three-chamber long-axis view and best visualized on imaging sequences with longer echo times (TEs).

**Figure 3:**
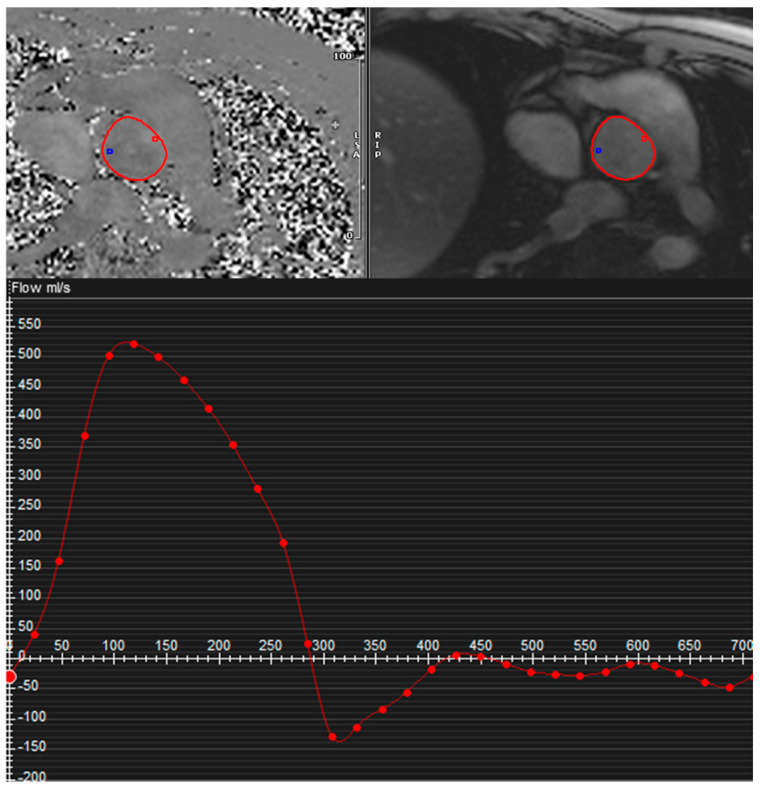
AR quantification by phase-contrast CMR: phase-contrast sequence (**above**) and aortic flow curve (**below**).

AR can be easily quantified using phase-contrast sequences perpendicular to the AAo across the sinotubular junction (Figure 3). The through flow, both forward and reverse, should be measured above the aortic valve and is normally expressed as:

Regurgitant volume (RV): mL/cardiac cycle

Regurgitant fraction (RF): % (proportion of forward flow through the Aortic Valve that returns to the LV) = (RV/aortic forward flow in systole) × 100%

CMR quantification may slightly underestimate the true volume of AR, due to the motion of the valve annulus during the cardiac cycle. In fact, in systole there is an increasing gap between the valve and the image plane for flow mapping due to the movement of the aortic valve towards the apex (particularly in vigorously contracting LV, as common in the context of severe AR) and to the elastic expansion of the aortic sinuses and root (particularly in cases of dilated Valsalva sinuses).

To avoid the aforementioned issue, the image plane for flow should be positioned as close to the valve as possible and, where available, “slice tracking” should be selected in order to obtain the flow image plane to move with the valve annulus.

However, it is important to keep in mind that BAV patients usually present with extremely eccentric AR jets, associated with complex flow patterns in the AAo, which may compromise the accuracy of flow measurement by phase-contrast imaging. In these cases, flow measurement in the AAo may lead to more pronounced underestimation of the forward flow, and for this reason, measurement in the LVOT or the aortic valve, where complex flow is less prominent, should be preferred in order to accurately quantify the systolic forward flow [30].

Finally, in a case in which phase-contrast images are considered unreliable, AR may still be quantified using the difference in stroke volume (SV) between the LV and right ventricle. In fact, when no other regurgitant valve is present, the difference in SV between the two ventricles should equal the AR. This method may also be used to corroborate the flow data if necessary.

### 3.2. LV Size, Function, and Fibrosis Evaluation

Longstanding, significant AR may result in LV chamber enlargement and systolic dysfunction. These morphological/functional data are extremely important to obtain, as they guide timing of intervention. In this scenario, CMR is able to provide detailed and reproducible assessments of both LV volumes and function.

A reliable measurement of ventricular volumes is also required to calculate the RV as the difference between LV-SV and RV-SV in those cases in which direct quantification of AR is not possible.

The analysis of LV volumes by CMR is based on the Simpson’s method of disks. A stack of SSFP Short Axis cine images from the base of the LV to the apex is acquired. The end-diastolic and end-systolic volumes are obtained by contouring the endocardial borders on a per-slice basis. The mitral valve annulus is conventionally used as landmark which separates the LV from the left atrium [32].

Following the functional study, tissue characterization is of paramount importance in order to identify areas of myocardial fibrosis. The Late Gadolinium Enhancement (LGE) technique is based on acquisition of dedicated sequences ten minutes after the administration of a 0.1–0.2 mmol/kg gadolinium contrast agent injection. Inversion recovery (IR) or phase sensitive inversion recovery (PSIR) sequences are the most commonly used; these are ECG-gated on mid to late diastole and usually acquired in the 2C, 3C and 4C and short-axis orientations, corresponding to the SSFP cine slices. It has previously been demonstrated that pressure and volume overload associated with significant AR may impact on the long term growth of cardiomyocytes with addition of new sarcomeres in series and could result in both interstitial [33] and replacement fibrosis [34], translating in a multifocal patter of LGE on post-contrast CMR images. Emerging data in literature, suggest that the presence of LV myocardial fibrosis in patients with AR may likely be a marker of adverse remodeling, that may cause greater LV function deterioration and a worse prognosis after surgery [35].

### 3.3. Myocardial T1 Mapping and Strain Imaging

Novel CMR techniques are currently being investigated in the study of the effect of long-standing AR on LV mechanics and tissue composition. Given the evidence of increased fibrosis in AR ventricles, the role of T1 mapping in stratification of these patients has been investigated as well. According to a small study from Sparrow et al., post-contrast T1 values in segments with abnormal contraction of AR patients are prolonged compared to normal controls [36], thus confirming the hypothesis of increased LV interstitial fibrosis. Furthermore, in nine patients with severe AR who underwent surgical aortic valve replacement, the extent of histologic interstitial fibrosis was compared to extracellular volume fraction measured on 3T CMR, demonstrating a strong correlation between the two parameters [37].

In order to emphasize the presence and impact of fibrosis on ventricular function in a subclinical stage when LVEF is still in normal range, Ungacta et al. investigated the role of Strain imaging in identifying abnormal patterns. They found a decrease in posterior wall circumferential strain in patients with AR six months after valve replacement [38]. These results have been confirmed in a recent study from Fernández-Golfín et al., demonstrating significant differences in GLS values between controls and AR patients and among AR severity groups [39]. In summary, emerging data suggest the key role of Strain imaging in helping the clinicians to identify LV dysfunction at a pre-clinical stage, thus possibly guiding the timing of intervention and specifically anticipating it before the detection of overt and irreversible effects of significant AR on LV function, morphology, and tissue characteristics.

### 3.4. Aortic Stiffness

The assessment of aortic stiffness, as measurement of distensibility or velocity of flow propagation, has developed special interest in order to predict progressive dilation of aorta. The stiffness of the thoracic aorta can be estimated by two non-invasive methods: measuring the velocity of propagation of flow (pulse wave velocity) or the change in diameter/area due to pressure pulse (distensibility). Aortic distensibility can be calculated as a change in cross-sectional area in CMR or diameter in echocardiography during the cardiac cycle divided by local pulse pressure, and it is considered as a marker of aortic elasticity and is inversely correlated with wall stiffness.

In many studies, aortic distensibility values were comparable in patients with BAV and normal subjects for similar aortic diameters. However, it is unclear whether the reduction in aortic distensibility with increasing diameter [40] is due to increased aortic stiffness or to other factors such as altered wall stress distribution caused by change in shape and pressure during dilation [41].

Pulse wave velocity (PWV), measured by phase-contrast CMR (PC-CMR), is the other method frequently used to evaluate aortic stiffness.

### 3.5. 4D Flow and Emerging Parameters

BAV morphology influences aortic flow patterns which in turn lead to different aortopathy phenotypes. According to numerous studies, the flow in the aorta of BAV patients is asymmetric, with velocities not being higher at the center of the vessel and exhibiting a direction angle greater than normal relative to the center of the aorta; all this leads to the formation of vortices [42]. Flow asymmetry and directional abnormalities can be assessed using normalized flow displacement and jet angle, respectively, while vortices can be quantified by measuring circulation (also known as in-plane rotational flow), systolic reversal flow, and more advanced metrics. These flow abnormalities are believed to induce changes in wall shear stress, the tangential force per unit area exerted by blood on the aortic wall. Flow irregularities have been observed in non-dilated BAV aortas, indicating that these issues are not merely secondary effects of dilation.

Thanks to the use of 4D flow MRI, it has been demonstrated that different fusion phenotypes cause different flow patterns: R/L cusp fusion results in an anteriorly distributed flow, while R/N fusion causes a predominantly posterior flow at the sinotubular junction that shifts to an anterior or right anterior in AAo [40]. Partial aortic valve leaflet fusion may also alter aortic flow patterns, leading to aorta dilation [43].

The use of 4D flow MRI in clinical practice appears to be promising in the quantification of eccentric regurgitation jets in BAV patients and in early identification of related complications.

## 4. Assessment of Chronic Aortic Regurgitation in BAV: Usefulness and Limitations of Cardiac-CT

Among imaging techniques, multidetector computed tomography (MDCT) plays an important role in the evaluation of patients with BAV and severe aortic regurgitation. A major benefit of CT is its superior spatial resolution. Besides coronary artery assessment, evaluation of anatomy of aortic valve and diameters of ascending and thoracic aorta, MDCT provides the opportunity to quantify LV systolic function and has been recently validated against echocardiography for quantifying the severity of aortic regurgitation. The ECG gating CT technique (using a retrospective or prospective technique) reduces cardiac motion artefacts, allowing for non-invasive coronary artery evaluation and cardiac morphological assessment. MDCT use is limited by exposure to potentially harmful radiation, and by the risk of inducing contrast nephropathy [44] in patients with pre-existing renal dysfunction. Other current limitations of MDCT are its relatively low temporal resolution (75–180 ms) and inability to directly quantify the transvalvular flow velocity.

### 4.1. Aortic Root and Valve Evaluation

**Figure 4:**
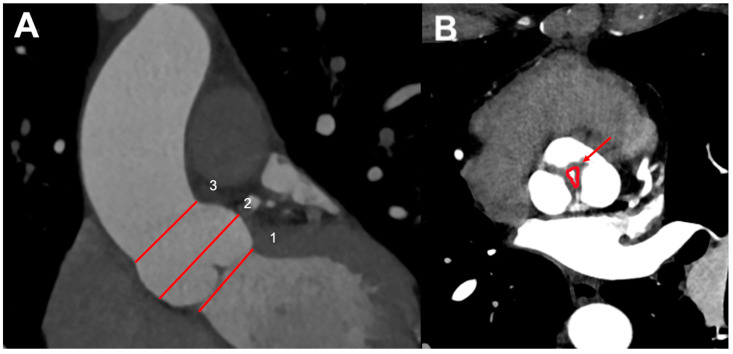
Measurements of aortic root at different levels by CT (**A**): (1) aortic valve annulus (the hinge points of the valve cusps); (2) midpoint of the sinuses of Valsalva; and (3) sinotubular junction. (**B**) CT planimetric measurement of aortic regurgitant orifice (ARO) (arrow).

Aortic root dimensions are conventionally measured, from an “optimized” sagittal oblique LVOT reconstruction in the mid-diastolic (prospective ECG gating) or end-diastolic phase (retrospective ECG gating), at three levels: (I) aortic valve annulus (where the valve cusps hinge); (II) midpoint of the sinuses of Valsalva; and (III) sinotubular junction (Figure 4A) [45]. Once the aortic diameters have been evaluated based on gender and body habitus (comparing with the reference ranges [46]), the aortic valve is assessed: number of leaflets (tricuspid, bicuspid, etc.), valvular calcification and free edge thickening [47].

On retrospective ECG-gated studies leaflet, the excursion can be visualized on a cine loop reconstruction in the cross-sectional valve plane. When an intrinsic valve disease is present, the leaflets are often retracted, thickened, and calcified; conversely, when AR is secondary to aortic root distortion, the leaflets may have a normal appearance but fail to co-apt centrally, leaving a regurgitant orifice in the diastolic phases [48]. In MDCT, the lack of cusp co-aptation can be better visualized in the end-diastolic phase of the cardiac cycle (reconstructed images approximately at 70% of the R-R interval) where it is possible to directly measure the maximum anatomic aortic regurgitant orifice (ARO) quantifying the severity of AR; several studies showed a good correlation between CT-derived ARO and echo-derived EROA for moderate and severe AR [49].

However, evaluation of ARO using MDCT should be carefully assessed in patients presenting eccentric AR or aortic valve cusp prolapse. The study by Davinder S. Jassal at al. showed a strong correlation between ARO by 64-MDCT and the individual quantitative TTE parameters of vena contracta, ratio of jet to LVOT height, and ratio of jet to LVOT cross-sectional area and demonstrated that MDCT has been demonstrated to detect the presence of AR with high specificity, positive predictive value, and diagnostic accuracy [49]. In conclusion, using planimetric measurements of the ARO area, MDCT is able to quantitatively evaluate AR severity.

### 4.2. LV Assessment

The dimensions of the left ventricle, measured in the end-diastolic phase, should also be routinely evaluated in the CT examination. Normal values are <12 mm for the septum, <8 mm for the lateral wall, and <6 cm for the end-diastolic diameter [50]. Ejection fraction should be calculated using retrospective datasets, typically achieved with vendor-specific contouring algorithms that map the endocardial borders and measure end-diastolic and end-systolic volumes.

## 5. Conclusions

AR assessment and grading estimation in BAV patients could be challenging because of irregularity of regurgitant jet and advantages and limitations of each imaging technique (Table 1, Table 2 and Table 3). Echocardiography remains the first-line imaging method thanks to a multiparametric evaluation, but in case of doubt due to the impossibility of using some parameters (such as the vena contracta, EROA, etc.) or the acoustic window, CMR and MDCT can play an important role, also allowing for a more detailed assessment of aortic and LV dimensions. A multi-imaging approach involving echocardiography, cardiac MRI, cardiac CT, and novel applied advanced technologies could provide a more comprehensive evaluation of AR, LV involvement and tissue characterization, and early identification of related complications, thus possibly giving a relevant contribution in guiding therapy and address appropriate patients’ management.

## Figures and Tables

**Table 1:** Advantages and limitations of echocardiographic assessment in BAV AR.

	Advantages	Limitations
Echocardiography	Widely available Cheap No radiation exposure No contrast agents required Information about aortic valve anatomy Visual estimation of AR jet and information about jet eccentricity by color flow Doppler imaging Quantitative assessment and grading of AR Vena contracta evaluation appliable even in eccentric jets Diastolic flow reversal in the descending aorta by pulsed wave Doppler for evaluation of AR severity Information about LV and aortic size Speckle tracking echo provides info about LV deformations in multiple directions and aortic distensibility Three-dimensional echo allows for visualization of the actual shape of the regurgitant aortic orifice	Operator- and window-dependent Irregular shape of the regurgitant orifice and eccentric AR jets in BAV limit quantitative assessment of AR and accuracy in AR grading The ratio between the regurgitant jet width and LV outflow tract diameter is not applicable for irregular-shaped orifices Vena contracta evaluation is not applicable in case of multiple jets The proximal isovelocity surface area method is limited by low feasibility because of difficulty in detection of the flow convergence zone and possible interposition of valve tissue Pressure half time requires adequate Doppler angle and beam alignment; thus, it is hardly applicable in eccentric jets

**Table 2:** Advantages and limitations about cardiac magnetic resonance (CMR) assessment in BAV AR.

	Advantages	Limitations
CMR	Acoustic window limitations Multiple imaging planes Accurate and reproducible Visualization of aortic valve anatomy Ventricular volumes/function assessment without geometrical assumptions Qualitative assessment of AR Accurate quantitative assessment of AR (also for eccentric jets) Visualization of aorta in toto Identification of associated abnormalities Detection of myocardial fibrosis	Not widely available Claustrophobia Difficulties in breath-holding Longer time of acquisition Compromised quality in case of arrhythmias Lower temporal resolution Quantitative assessment of aortic regurgitation should be carried out in the LVOT or aortic valve plane when extremely eccentric AR jets are present

**Table 3:** Advantages and limitations of cardiac CT assessment in BAV AR.

	Advantages	Limitations
Cardiac CT	No body habitus/acoustic window limitations Multiple imaging planes Accurate and reproducible High spatial resolution Visualization of aortic valve anatomy Visualization of aorta in toto Identification of associated abnormalities Planimetric measurements of the ARO Ventricular volumes/dimensions assessment Optimal visualization of valve calcification	Use of iodinated contrast (risk of contrast induced nephropathy and allergy) Use of ionizing radiations No qualitative assessment of AR ARO is the only quantitative parameter to be used

## Data Availability

No new data were created or analyzed in this study. Data sharing is not applicable to this article.

## Abbreviations

BAV	bicuspid aortic valve
AR	aortic regurgitation
AAo	ascending aorta
TTE	transthoracic echocardiography
CMR	cardiac MRI
LVOT	left ventricle outflow tract
L	left
R	right
N	non coronary
CoA	coarctation
LV	left ventricular
EROA	effective regurgitant orifice area
RV	regurgitant volume
LVEF	left ventricular ejection fraction
PWV	pulse-wave velocity
PC-CMR	phase-contrast CMR
STE	speckle-tracking echocardiography
GLS	global longitudinal strain
RF	regurgitant fraction
SV	stroke volume
LGE	late gadolinium enhancement
MDCT	multidetector computed tomography
ARO	aortic regurgitant orifice
